# Awareness and use of evidence based medicine (EBM) in medical trainees

**Published:** 2014-07

**Authors:** MAHDIEH MOMAYYEZI, HOSSEIN FALLAHZADEH, MOHAMMAD MOMAYYEZI

**Affiliations:** 1 Research center of prevention and epidemiology of non-communicable disease, public health school, Shahid Sadoughi University of Medical Sciences, Yazd, Iran;; 3 School of Medicine, Shahid Sadoughi University of Medical Sciences, Yazd, Iran

## Dear editor

The concept of evidence based medicine (EBM) involves a systematic approach for integration of best available research evidence into medical decision making for physicians, medical trainees and researchers. The EBM process includes five essential steps: formulating the questions, searching for evidence, appraising the evidence, and applying and evaluating the results (1). In a descriptive study, the data about EBM skills obtained from the medical trainees showed that printed textbooks served as the first source of information in 80% of cases and followed by E-books as the second source. The hard copy journals in libraries had the lowest use. Easy access to information was selected as the main reason for using printed resources. Also, fast access to information was the most important reason for using electronic resources. In a study conducted by Rohani, it was found that physicians used printed sources more than e-books (2). This was due to their easy access to the printed reference books. Considering the fact that it takes a lot of time for new medical information to log in into the reference books, effective treatment of a disease may be entered in printed sources after its efficiency is confirmed.

45.2% of the participants were familiar with INLM. 32.1% of the students had attended the training course on INLM. 85.7% of the participants were familiar with the PubMed, 28.6% with the Elsevier and 16.7% with Science direct. 31% of the participants were familiar with the concept of EBM, but 7.1% were aware of the resources of EBM, and 12.2% had attended the special courses on EBM. The results of the recent data taken from internet showed that 53.8% of participants sometimes used it in diagnosis and treatment process and 28.2% of them used it after being developed into textbooks. Also, this information was sometimes useful for 75.8% of participants.

Half of the students were familiar with the concept of EBM, but a few had attended the introductory course on EBM. Hanson in his study maintained that only 1.9% of physicians used specific methods to find valid evidence of medicine (3). Taheri showed that medical students were satisfied with the workshop on EBM and stated that familiarity with EBM was essential for proper functioning (4). Therefore, it seems necessary to organize the training workshops on the concepts of EBM as a part of formal training for medical trainees to change the students' attitudes.

## References

[1] Akobeng AK (2005). Principles of evidence based medicine. Arch Dis Child.

[2] Rohani A, Akbari V, Mordian K (2012). Assessment of information about evidence base medicine in specialist and family physicians of Yasooj University of medical sciences. Iranian Journal of Medical Education.

[3] Hanson B, Bhandari M, Audige L, Helfet D (2004). The need for education in evidence-based orthopedics: an international survey of AO course participants. Acta Orthop Scand.

[4] Taheri H, Mirmohammad Sadeghi, Adibi I, Ashourioun V, Sadeghizadeh A, Adibi P (2006). The effect of an evidence based medicine workshop on undergraduate medical students’ skills in applying EBM. Iranian Journal of Medical Education.

